# Off label use of Aripiprazole shows promise as a treatment for Myalgic Encephalomyelitis/Chronic Fatigue Syndrome (ME/CFS): a retrospective study of 101 patients treated with a low dose of Aripiprazole

**DOI:** 10.1186/s12967-021-02721-9

**Published:** 2021-02-03

**Authors:** L. D. Crosby, S. Kalanidhi, A. Bonilla, A. Subramanian, J. S. Ballon, H. Bonilla

**Affiliations:** 1 Stanford University School of Medicine, Stanford, USA; 2 University of Michigan, College of Literature, Sciences, and Arts, Ann Arbor, MI USA

Myalgic Encephalomyelitis/Chronic Fatigue Syndrome (ME/CFS) is a chronic, debilitating illness of unknown etiology. An ME/CFS diagnosis is based solely on symptoms with case definitions made by expert consensus, including the Fukuda (1994), Canadian Consensus Criteria (CCC, 2003), International Consensus Criteria (ICC, 2011), and the Institute of Medicine (IOM, 2015) case criteria. According to the most recent IOM case definition, the core symptoms of ME/CFS include debilitating fatigue, unrefreshing sleep, post-exertional malaise, and either cognitive dysfunction or orthostatic intolerance [1]. Although the cause of the illness is unknown, a growing body of evidence suggests that ME/CFS involves inflammation of the brain. Up to 85% of patients with ME/CFS report symptoms of cognitive impairment also referred to as “brain fog,” which includes difficulty with memory, attention, and information processing. Additional evidence includes changes in inflammatory cytokines in both plasma and cerebrospinal fluid correlated with the severity of symptoms [2]. Other studies using positron emission tomography (PET) show evidence of activated microglia or astrocytes in various regions of the brain in patients with ME/CFS [3].

Dopamine D2 receptor agonists have been shown to mediate neuroinflammation, microglial activation, and cell death in animal models and humans [4–6]. This suggests that dopamine-modulating drugs like aripiprazole may lead to clinical improvement in fatigue and cognitive symptoms in ME/CFS. Given the lack of approved drugs for treating this condition, we were interested in exploring the potential benefit of low doses of aripiprazole in our Stanford University ME/CFS clinical practice.

In a retrospective study, we reviewed the medical records of 101 patients who met the criteria for a ME/CFS diagnosis according to three separate case definitions (Fukuda, CCC, and IOM) and who received off-label aripiprazole (Table 1). Medical records were included for individuals evaluated in the clinic at least twice, representing periods before and after the use of the medication. The age range was from 18 to 84 years old (mean 50 years), with a gender distribution of 67% female and 33% male, and the duration of illness was from 1 to 54 years (median 13 years). The daily oral dose of aripiprazole ranged from 0.2 to 2.0 mg/day (mean 1.1 mg/day). Dosage started at 0.25 mg/day and titrated up or down based on each patient's observations and feedback. The duration of aripiprazole therapy ranged from less than one month to 17 months (mean 7.8 months). Patient records were also evaluated for concurrent use of various classes of antidepressants, including selective serotonin reuptake inhibitors (SSRI), serotonin-norepinephrine reuptake inhibitors (SNRI), serotonin modulators, norepinephrine–dopamine reuptake inhibitors (NDRIs), and tri-cyclic antidepressants. The difference in antidepressant use between responders vs. non-responders was not statistically significant (*p* = 0.145) using the test for proportions, suggesting that antidepressant use does not predict or preclude a clinical response to aripiprazole.

**Table 1:** Demographics for individuals with ME/CFS taking aripiprazole (Abilify)

Demographics	Responders	Non-responders	Total
Individuals with ME/CFS, n (%)	75 (74.3%)	26 (25.8%)	101 (100%)
Mean age, years (range)	48.9 (18–76)	52.2 (28–84)	49.7 (18–84)
Duration of illness, years (range)	12.6 (2–54)	15.8 (1–44)	13.4 (1–54)
Female, n (%)	52 (69.4%)	16 (61.5%)	68 (67%)
Male, n (%)	23 (30.6%)	10 (38.5%)	33 (33%)
Aripiprazole dose, mg (range)	1.2 (0.25–2.0)	0.7 (0–2.0)	1.1 (0–2.0)
Duration of treatment, average months (range)	8.0 (0–17)	7.1 (0–13)	7.8 (0–17)
Concurrent antidepressant use	*46,175* (61.3%)*	11/26 (42.3%)*	57/101 (56.4%)

^*^p = 0.145

During each clinic visit, patients were asked to rate their symptoms on a scale of 0–10. Lower values represented milder illness, and “0” represented the absence of the symptoms. As this was a retrospective study, some records from patients contained descriptive statements (e.g., feeling “better” or “worse”), or had missing values for one or more symptoms. Thus, symptom scores were calculated based on the number of individuals with complete records for each symptom. We analyzed each group separately to understand the magnitude of change in symptom scores between responders and non-responders. Statistical analysis was performed using the nonparametric Wilcoxon Signed Rank Test. This method was used because the dataset included survey results with ordinal data in matched pairs (before and after), and it does not require assumptions about the underlying distribution.

Results: Of the 101 patients taking aripiprazole, 75/101 (74%) experienced an improvement in one or more categories: fatigue, brain fog, unrefreshing sleep, and frequency of post-exertional malaise (PEM) episodes, or “crashes.” Twelve individuals (12%) had no observable difference in symptoms at the maximum dose of 2 mg, and 14 individuals (14%) reported worsening of symptoms or onset of side effects that led to discontinuation of the drug (Fig. 1).

**Fig. 1:**
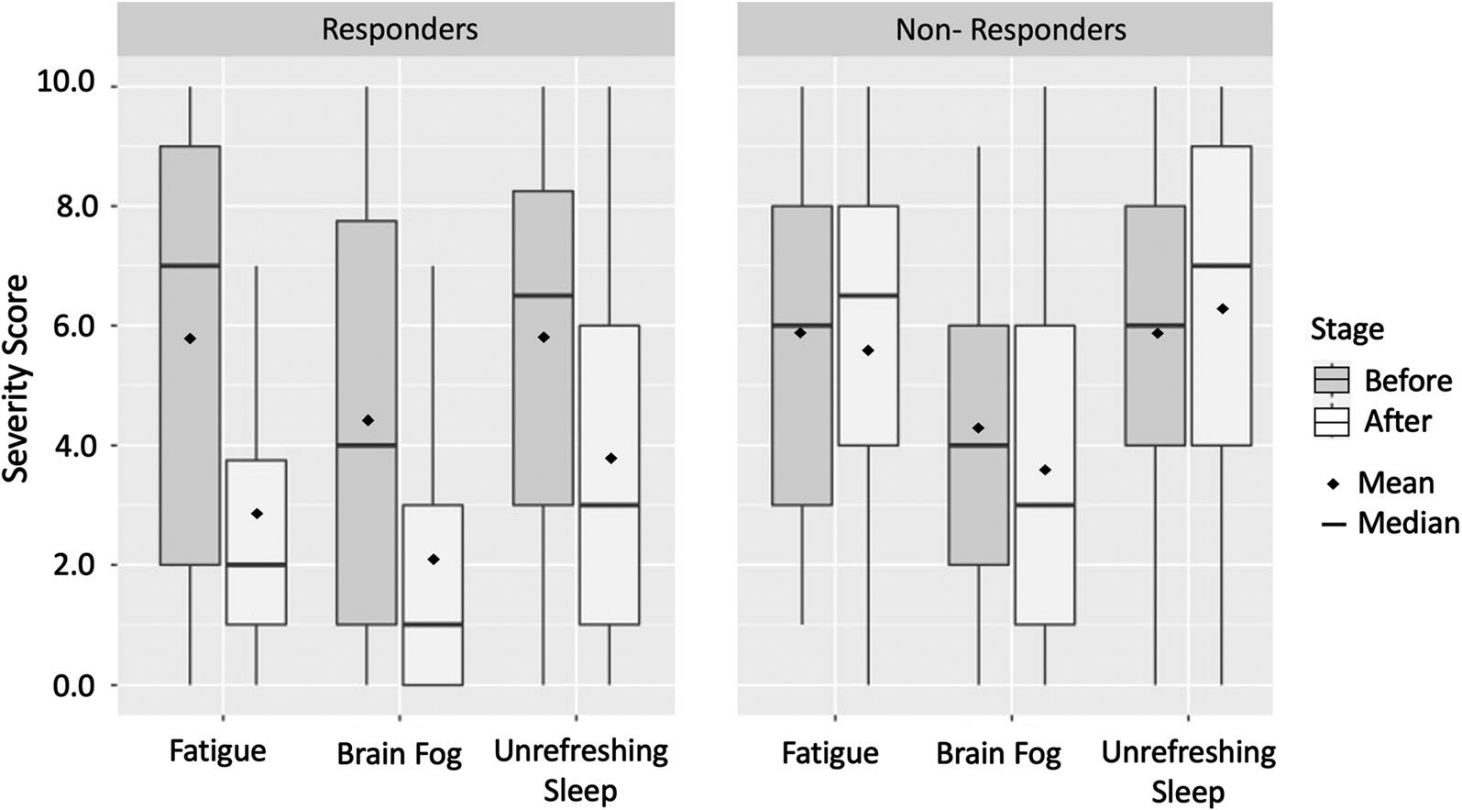
Changes in symptom severity scores between Responders and Non-Responders taking aripiprazole. Symptoms were rated on a scale of 0 (no symptom) to 10 (worst). For responders, average fatigue score changed by − 2.89 units (*p* < 0.001), brain fog changed by − 2.33 units (*p* < 0.001), and unrefreshing sleep changed by − 2.05 units (*p* < 0.001). Non-Responders showed non-significant differences of − 0.2 units for fatigue, (*p* = 1), − 0.65 units for brain fog (*p* = 0.289), and = 0.42 for unrefreshing sleep (*p* = 0.4105)

Among the responders, the average fatigue score changed from 5.76 to 2.86 (n = 66, delta = − 2.89, *p* < 0.001). Similarly, the average brain fog score reduced from 4.39 to 2.06 (n = 66, delta = − 2.33, *p* < 0.001), and sleep quality ratings changed from 5.80 to 3.75 (n = 60, delta = − 2.05, *p* < 0.001). Post-exertional malaise was recorded as the time interval, in days, between the onset of each PEM the episode, which was converted to occurrence per day, or 1/(time interval between episodes). The absence of PEM was recorded as “0.” For responders, the average frequency of PEM was 0.24 occurrences per day (or every 4.2 days) before taking the medication and 0.12 occurrences per day (every 8.3 days) while taking aripiprazole (n = 59, *p* < 0.001). Fifty-seven individuals qualitatively rated the episodes of PEM as milder and shorter in duration and 18 individuals reported a complete resolution of PEM with no new episodes. Improvements in the quality of life enabled six patients to return to work, and four patients reported improvement of their movement disorder (myoclonic dystonia).

For non-responders, the average fatigue score was 5.85 before and 5.65 after taking aripiprazole (n = 20, delta = − 0.2, *p* = 1), whereas average brain fog scores went from 4.25 to 3.60 (n = 20, delta = − 0.65, *p* = 0.289). Sleep quality was rated at 5.89, which increased non-significantly to 6.32 after taking aripiprazole (n = 19, delta = 0.42, *p* = 0.4105). The frequency of PEM episodes showed no statistically significant change at 0.23 per day (4.3 days) before aripiprazole to 0.25 per day (4.0 days) afterwards (*p* = 0.196). Qualitatively, non-responders reported that their PEM frequency and severity stayed the same but did not worsen while taking aripiprazole.

Patients were asked to report any side effects via a secure communication portal or during an office visit. Beyond the initial titration phase, side effects included headache (5 individuals), irritability/agitation (4 individuals), insomnia (3 individuals), and two individuals reported extreme agitation. Bodyweight was also recorded at each office visit before and after taking aripiprazole. On average, patients gained 0.15 kg per month while taking aripiprazole (*SD* 0.65 kg/month, max = 1.51 kg/month, min = − 2.36 kg/month). Ten individuals gained between 5.0 and 12.0 kg in total and eight individuals lost substantial weight, ranging from − 6.0 to − 14.0 kg. Given the potential for confounding variables (e.g., changes in diet or activity levels), we could not attribute changes in body mass to the use of the medication.

In summary, the number of positive responders in a group of 101 patients taking aripiprazole was significantly greater than the number of patients who did not respond or had negative experiences. Also, the magnitude of perceived improvement was significant. Some patients failed to observe any benefit, and a small subset of patients experienced side effects that required the medication to be discontinued. Overall, these results suggest that aripiprazole may effectively reduce symptoms of ME/CFS and warrants further investigation in a randomized clinical trial. Exploring the mechanism of action for aripiprazole in neuroinflammatory conditions may also provide new insight into the pathogenesis of ME/CFS.

## Data Availability

The data of this study has been stored for future reference and available upon request.
