# Publisher Correction to: Off label use of Aripiprazole shows promise as a treatment for Myalgic Encephalomyelitis/Chronic Fatigue Syndrome (ME/CFS): a retrospective study of 101 patients treated with a low dose of Aripiprazole

**DOI:** 10.1186/s12967-021-02875-6

**Published:** 2021-05-21

**Authors:** Journal of Translational Medicine

**Affiliations:** London, UK

## Publisher Correction to: J Transl Med (2021) 19: 50 10.1186/s12967-021-02721-9

During the publication process an error was introduced in the original publication of this article [1].

In Table 1 the value for Concurrent antidepressant use was accidentally changed. In this correction article the incorrect and correct values are published for clarification. The full table is available via the original article. The publisher apologizes to the readers and authors for the inconvenience. The original article has been updated.

## Incorrect

**Table:** 

Demographics	Responders
Concurrent antidepressant use	*46,175* (61.3%)*

*p=0.145

## Correct

**Table:** 

Demographics	Responders
Concurrent antidepressant use	46/75 (61.3%)

*p=0.145

## References

[1] Crosby LD, Kalanidhi S, Bonilla A, Subramanian A, Ballon JS, Bonilla H (2021). Off label use of Aripiprazole shows promise as a treatment for Myalgic Encephalomyelitis/Chronic Fatigue Syndrome (ME/CFS): a retrospective study of 101 patients treated with a low dose of Aripiprazole. J Transl Med.

